# Resilin and chitinous cuticle form a composite structure for energy storage in jumping by froghopper insects

**DOI:** 10.1186/1741-7007-6-41

**Published:** 2008-09-30

**Authors:** Malcolm Burrows, Stephen R Shaw, Gregory P Sutton

**Affiliations:** 1 Department of Zoology, University of Cambridge, Cambridge, CB2 3EJ, UK; 2 Department of Psychology, Dalhousie University, Halifax, Nova Scotia, Canada

## Abstract

**Background:**

Many insects jump by storing and releasing energy in elastic structures within their bodies. This allows them to release large amounts of energy in a very short time to jump at very high speeds. The fastest of the insect jumpers, the froghopper, uses a catapult-like elastic mechanism to achieve their jumping prowess in which energy, generated by the slow contraction of muscles, is released suddenly to power rapid and synchronous movements of the hind legs. How is this energy stored?

**Results:**

The hind coxae of the froghopper are linked to the hinges of the ipsilateral hind wings by pleural arches, complex bow-shaped internal skeletal structures. They are built of chitinous cuticle and the rubber-like protein, resilin, which fluoresces bright blue when illuminated with ultra-violet light. The ventral and posterior end of this fluorescent region forms the thoracic part of the pivot with a hind coxa. No other structures in the thorax or hind legs show this blue fluorescence and it is not found in larvae which do not jump. Stimulating one trochanteral depressor muscle in a pattern that simulates its normal action, results in a distortion and forward movement of the posterior part of a pleural arch by 40 μm, but in natural jumping, the movement is at least 100 μm.

**Conclusion:**

Calculations showed that the resilin itself could only store 1% to 2% of the energy required for jumping. The stiffer cuticular parts of the pleural arches could, however, easily meet all the energy storage needs. The composite structure therefore, combines the stiffness of the chitinous cuticle with the elasticity of resilin. Muscle contractions bend the chitinous cuticle with little deformation and therefore, store the energy needed for jumping, while the resilin rapidly returns its stored energy and thus restores the body to its original shape after a jump and allows repeated jumping.

## Background

Movements that are both fast and powerful must overcome the constraints imposed by the properties of striated muscle. A muscle can either contract rapidly and generate limited energy, or it can contract slowly to generate its maximal energy. The balance between these two extremes sets a functional limit to the amount of power (energy/time) that a muscle can generate [1,2]. Animals that use movements demanding both high speed and power have to overcome these limitations by slowly deforming elastic structures to maximise the energy stored, and then deliver this stored energy by rapid recoil [2-4]. In this study, we analyse the nature and action of specialised structures that store and release energy to power the most effective jumping insect so far described, the froghopper [5,6].

Arthropods often use deformation of their exoskeleton as an elastic energy store, particularly when generating fast and powerful predatory strikes or when jumping. To strike a prey, a mantis shrimp first locks specific joints of its raptorial limbs and co-contracts the muscles to deform particular regions of its exoskeleton. Once this is deformed, one of the muscles relaxes [7] and recoil of the exoskeleton powers the strike in 5 ms [8]. Similarly, a trap-jaw ant powers its jaw strike by bending the cuticle of the head, the recoil of which then delivers the strike in less than 1 ms [9].

Locusts jump rapidly and powerfully by co-contracting the flexor and extensor tibiae muscles of their enlarged hind legs for a sustained period [10-12]. Approximately 50% of the required energy is stored by bending two cuticular semi-lunar processes at the femoro-tibial joint of each hind leg. Recoil of these compliant processes that are reinforced to withstand the high stresses placed upon them, delivers the stored energy rapidly to power jumping [13,14]. Click beetles jump rapidly by jack-knifing their bodies at the junction between the pro- and mesothorax as a result of elastically loading and then releasing the cuticle [15-17].

Many arthropods also store and release energy in the rubber-like protein resilin. It is found at many joints and tendons of arthropods where fast, repeated actions or elastic energy storage are required [18]. For example, cicadas have resilin in their sound-producing tymbals [19] and some can produce sharply resonant pulses of sound at 13 kHz [20]. Fleas, which use thoracic muscles for jumping, are suggested to store energy in a pad of resilin in the internal skeleton of the thorax [21-23]. Click beetles may also store some energy for jumping in resilin [24]. Resilin consists of coiled peptide chains linked together in a stable, isotropic, three-dimensional network by the fluorescent amino acids, dityrosine and trityrosine [25-27]. Resilin is mechanically highly deformable and shows almost perfect elastic recovery [28]. For example, the tendon of the pleuro-alar muscle of the dragonfly *Aeshna *can be stretched to twice its length for months without creep only to return rapidly to its original length once the load is removed [28]. Energy loss from resilin during movements at 200 Hz is less than 5% [29], suggesting that it can act as a useful spring over a wide range of speeds.

Resilin is colourless but can be revealed by exploiting its fluorescent properties accorded to it by its two tyrosine components [25], of which dityrosine appears to be more important [28] and most intensively studied [27]. Dityrosine, trityrosine and resilin each emit a uniform blue fluorescence when excited by ultraviolet (UV) light. Dityrosine and trityrosine as protein cross-links, impart fluorescence to structures such as the chorion of *Drosophila *eggs [30]. To determine if the blue fluorescence is from resilin, it is necessary to use a defining characteristic, which is that the excitation spectra are reversibly pH-dependent; the emission spectra are nearly identical and remain pH-invariant, with maxima near 420 nm [25]. At neutral to high pH, the near-UV absorption of dityrosine and trityrosine centres on 318 and 323 nm, respectively, declining gradually to zero near 370 nm. At acid pH, the absorption spectra of both amino acids shift to shorter wavelengths with a similar peak for both at 285 nm, with absorption now extending out only to about 330 nm [25,27]. With appropriate filters, therefore, it is possible to isolate the fluorescence of the high pH form of resilin from its low-pH alternative, so providing an identification signature for the protein [31].

Froghoppers (Hemiptera, Cercopoidea) are the most accomplished jumping insects which must store energy to achieve their jumping prowess. They use huge muscles in their thorax to move the trochantera of their hind legs and accelerate in less than 1 ms to take-off velocities of 4.7 m/second. The power requirements for this behaviour (10^6 ^W/kg), are well above the power that is available through direct muscle contraction (250 W/kg), indicating that an elastic energy store is required [2,32]. In a related lantern fly (Fulgoridae) that also jumps, the skeletal linkage between each hind coxa and the hinge of the corresponding hind wing, the pleural arch or Ugsprungsplatte [33,34], is enlarged and specialised. Suggested deposits of resilin have been reported in the internal thoracic skeleton of jumping planthoppers (Delphacidae) [23] and in the 'inner area of the pleural region' of froghoppers [35]. Thus, the pleural arches associated with the hind legs appear to be possible sites where energy could be stored for jumping in froghoppers and these cuticular structures may contain resilin.

We will show: (1) that the bilaterally paired pleural arches of froghoppers are bow-shaped composites of stiff chitinous cuticle and elastic resilin; (2) during jumping, these bows are deformed so that they have the potential to store energy; (3) the energy storage is primarily in the chitinous cuticle of the pleural arches, with the resilin adding other essential properties to these composite structures. A mechanism is therefore provided to explain the storage of energy that is essential for the powerful jumping of these insects.

## Results

**Figure 1:**
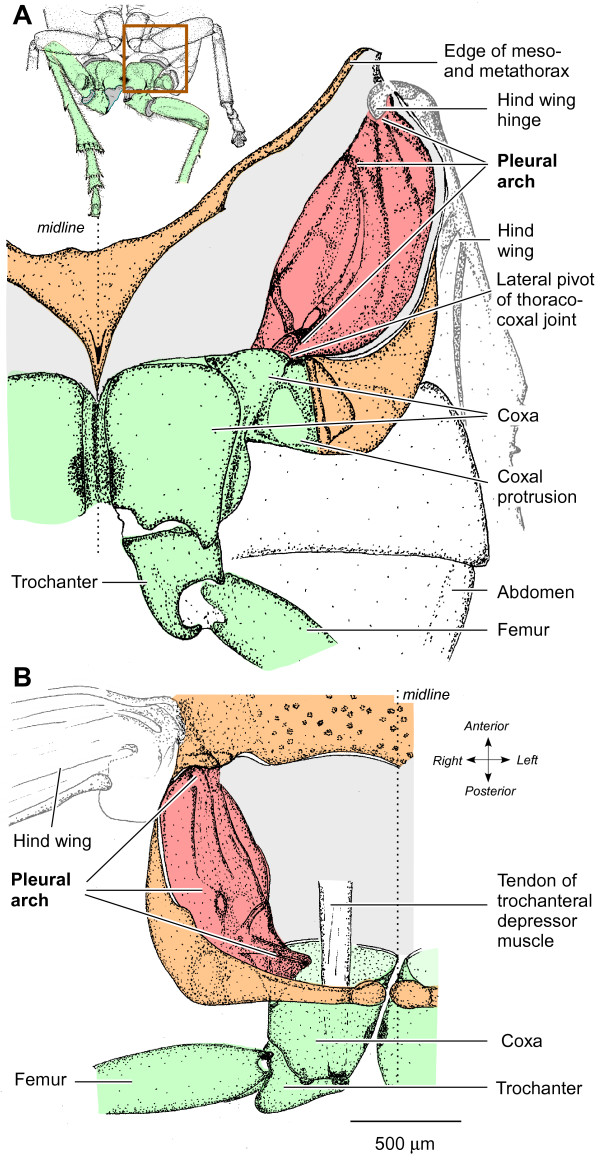
**Structure of the internal skeleton of the metathorax of *Aphrophora***. (A) Ventral view of the left half of the metathorax and proximal joints of the left hind leg. The box on the inset drawing indicates the area drawn in detail. The ventral cuticle of the thorax (orange) and the muscles were removed to reveal the pleural arch (red), linking the lateral edge of the coxa of the left hind leg (green) ventrally to the articulation of the left hind wing dorsally. (B) Dorsal view to show the left pleural arch curving ventrally from the hinge of the left hind wing to the lateral hinge of the thoraco-coxal joint of the left hind leg. The large tendon of the trochanteral depressor muscle is also shown.

The key movements of the hind legs of froghoppers in jumping occur at the joints between the coxae and trochantera [6]. The depression movements of the trochantera that propel a jump are generated by a pair of large and complex depressor muscles, each of which consists of four parts [36]. The two largest and one small part occupy most of the space in one half of the metathorax, while a fourth small part is restricted to a coxa. All four parts of one muscle insert on an impressively large tendon that runs through a coxa and inserts on the medial wall of a trochanter. In preparation for a jump, the muscle contracts for a few seconds without depressing its trochanter, but because the tendon inserts on the trochanter (Figure 1) and the fibres of the two large parts attach to the internal skeleton between the meso- and metathoracic segments, the coxa moves forwards. A jump occurs when the force generated by this contraction has reached a critical level and when mechanical 'latches' on the coxa and femur release, allowing the two trochantera to depress at the same time. What structures allow these prolonged and powerful contractions to occur without collapsing the thorax? Can they store the energy generated by the slow contraction of the muscle and deliver it rapidly to power the jump?

## Pleural arches

The two hind coxae are closely apposed at the midline and each has a prominent lateral part with a ventrally pointing protrusion (Figure 1A). A coxa can rotate about the metathorax by some 25° at two pivots, one at its anterior, ventral and lateral edge, and the second at its anterior, medial edge. The lateral pivot of each coxa is linked to the hinge of the ipsilateral hind wing by a large and complex pleural arch (Figure 1A and 1B). Each of these paired structures is 1.5 mm long, 0.7 mm wide and 0.5 mm deep (dorso-ventrally) and consists of large regions of translucent cuticle interspersed with ridges of sclerotised, dark cuticle. Ventrally, thin dark ridges of an arch converge on the lateral pivot with the coxa. Each arch curves outwards towards the lateral edge of the thorax and dorsally towards the hinge of a hind wing, giving an overall appearance of paired bows. The arches are large relative to those of the front and middle legs, which are not thought to provide much, if any, power for jumping [32].

## The pleural arches are a composite of resilin and chitinous cuticle

**Figure 2:**
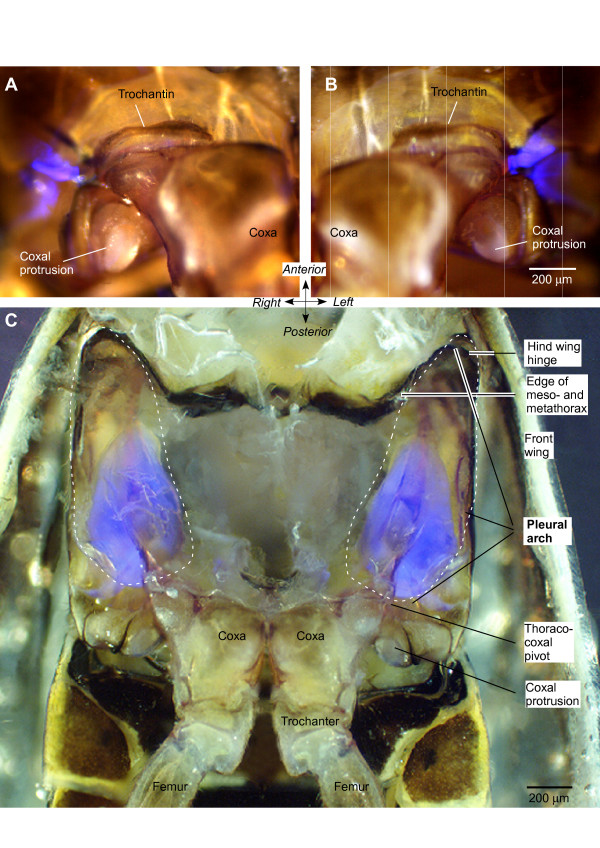
**Fluorescent structures in the metathorax of *Aphrophora *and *Philaenus***. Externally visible fluorescence at the right (A) and left (B) lateral pivot of the thoraco-coxal joints of the hind legs of *Aphrophora*. Bright field images and those illuminated with ultraviolet light (see Methods) at the same position and focal plane are superimposed. (C) Ventral view of the internal metathorax of *Philaenus *after removal of the ventral cuticle and thoracic muscles. The pleural arches linking the coxae with the hinges of the hind wings are outlined with dashed lines. Part of each pleural arch fluoresces bright blue.

In an intact froghopper, two bilaterally symmetrical patches of vivid blue fluorescence were revealed when the ventral surface of the metathorax, including the proximal segments of the two hind legs, was illuminated with UV light (Figure 2A and 2B). Each patch appeared to consist of two parts separated by a narrow non-fluorescent gap. One part, approximately 250 μm long and 100 μm wide, extended anterio-laterally from the pivot between the trochantin, the anterior and lateral edge of the coxa and the metathorax. The second was C-shaped with one arm extending anteriorly and laterally, and the other posteriorly and laterally. Both arms were about 250 μm long. The signal-to-noise ratio of the fluorescence at the thoraco-coxal pivot was high so that the outline of the structure was clearly delineated. No other blue fluorescence of comparable intensity was seen in the metathorax or in the hind legs. This pattern of fluorescence was consistent in all intact adult froghoppers of both *Aphrophora *and *Philaenus *examined. The soft articular membranes between the proximal joints of the hind legs, however, showed fainter fluorescence that was also seen with other filter sets, unlike that of the pleural arch.

**Figure 3:**
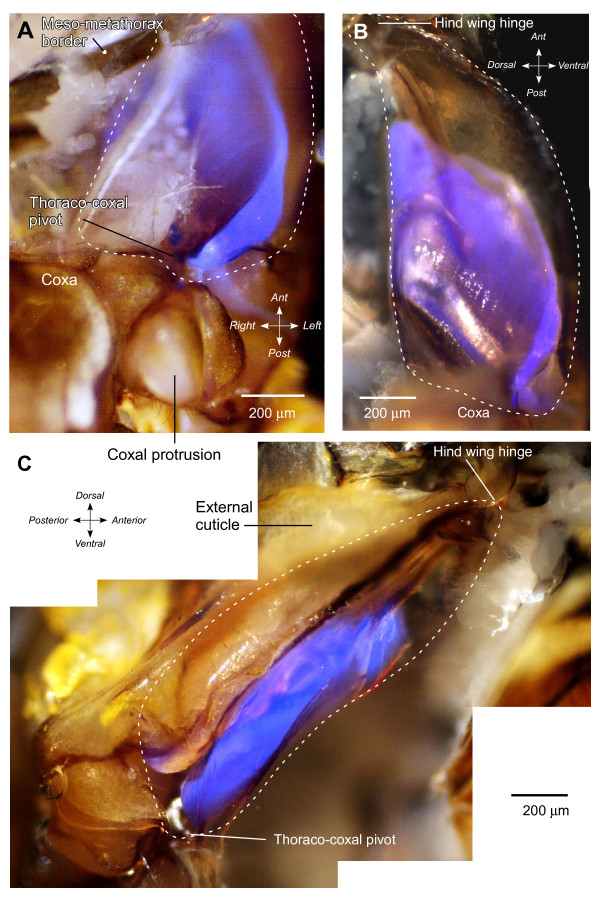
**Intense blue fluorescence in part of the pleural arch of the left half of the metathorax of *Aphrophora***. (A) Ventral view. The ventral cuticle and muscles of the left metathorax were removed, but the tracheae and some fatty tissue remain medially. (B) Medial view looking outwards after the metathorax had been split at the midline and muscles removed. (C) Lateral view from a montage of photographs. The lateral wall and muscles of the metathorax were removed. The dashed lines indicate the outline of the whole pleural arch. The fluorescent part is clearly curved antero-posteriorly (A and C) and dorso-ventrally (B and C).

Further exploration of the metathorax after superficial dissection showed that the externally visible fluorescence represented only a small part of a much larger and complex internal structure extending from the ventral surface dorsally (Figure 2C). A bow-shaped structure showing vivid blue fluorescence was revealed as part of the pleural arch after removal of the thin cuticle of the metathorax and the underlying muscles that move the trochanter and coxa. In *Aphrophora*, this fluorescent bow had a length of 1084 ± 20.1 μm (mean ± standard error of the mean, *N *= 5), a width of 504 ± 17.9 μm (*N *= 8), and a thickness of 127 ± 2.4 μm (*N *= 7). The externally visible part of this bow (Figure 2A and 2B) represents the thoracic part of the articulation with the coxa. From this point, the bow curves laterally and dorsally but ends some 430 μm short of the articulation with the hind wing (Figure 3A–C). The fluorescent part, therefore, represents some 70% of the total length of the internal skeletal structure, the pleural arch, between a hind coxa and the hinge of a hind wing.

These observations show that the bow of fluorescent material is an integral part of the pleural arch linking the coxa ventrally to the hinge of the hind wing dorsally and that the arch itself is a composite structure of fluorescent and non-fluorescent material (Figures 1, 2C and 3). The ventral end of the fluorescent material forms the lateral pivot with the coxa (thoraco-coxal joint), but its dorsal end does not extend as far as the articulation of a hind wing. The articulation with the wing hinge is, therefore, formed solely by the chitinous cuticular part of the pleural arch.

### Development of fluorescence in larvae

The larvae of *Philaenus *live a confined life enveloped by a froth made by blowing air into their urine. Unlike the free-living adults, the successive larval stages are unable to jump, but following the final moult, adults can jump within minutes of emerging from their froth. We therefore examined the fluorescence in larvae of different sizes to see if there was a correlation between the presence of fluorescence and jumping.

**Figure 4:**
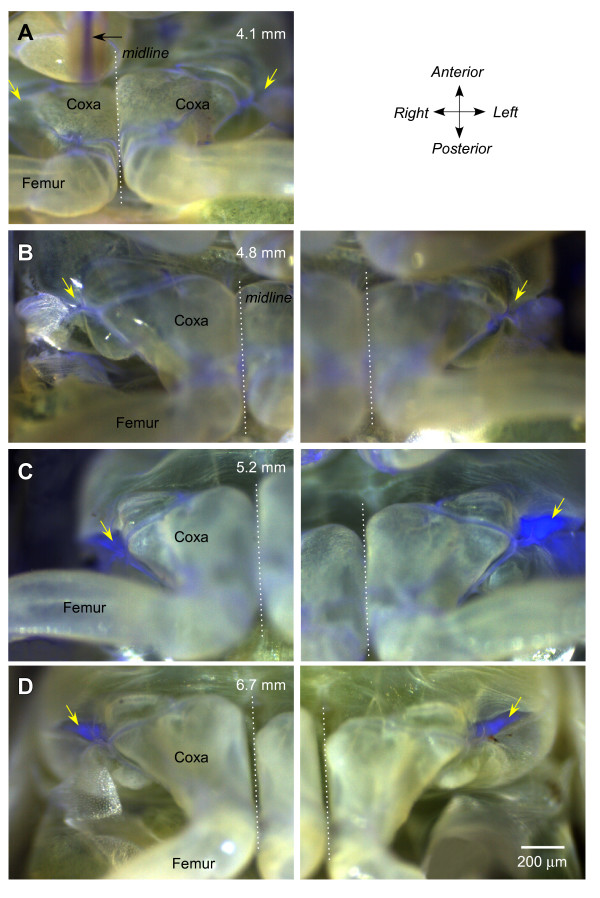
**Development of fluorescence during maturation of successive larval stages of *Philaenus***. (A)and (B) In two larvae with body lengths of 4.1 mm and 4.8 mm, there was no fluorescence at the thoraco-coxal joints as there was in adults (diagonal yellow arrows). Some fluorescence is apparent in the soft membrane between the proximal joints of the hind legs and in the mouthparts (horizontal black arrow in A). (C) A larger larva (5.2 mm body length) shows strong fluorescence at the thoraco-coxal joint of the left but little at the right hind leg. (D) In the larval stage (6.7 mm long) preceding the final moult to adulthood, fluorescence is present at both thoraco-coxal joints. In (A), the two hind legs are photographed in one image, in (B)-(D), they are represented in two images. The dotted lines indicate the midline.

In the smallest larvae that we examined, which measured 4 to 5 mm in body length, or less than half that of an adult, no fluorescence was found at the lateral pivot of the thoraco-coxal joint, even though the ventral cuticle was more transparent (Figure 4A and 4B). Fluorescence was, however, found in the soft articular membrane of the proximal joints of the hind legs and in the sucking mouthparts that projected as far posteriorly as the anterior edge of the hind coxae (Figure 4A). In larvae longer than 5 mm, fluorescence started to appear in the pleural arches. In some larvae, the intensity of the fluorescence was greater on one side than the other, indicating that the deposition process was underway but with some delay between the two sides (Figure 4C). Finally in late larval stages, the fluorescence in the pleural arches was intense and always present on both sides of the body (Figure 4D).

### Nature of the fluorescence

**Figure 5:**
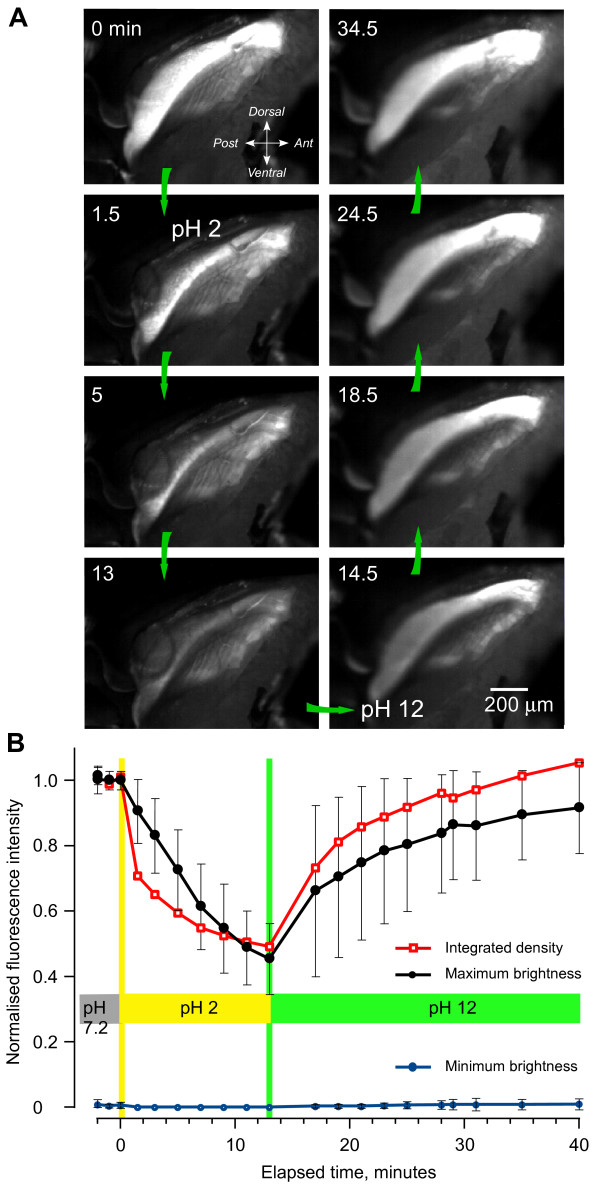
**Changes in the intensity of fluorescence with pH**. (A) Side view of the right half of the metathorax after the cuticular side wall and thoracic muscles were removed; the original colour images, taken at the times indicated, have been converted to black and white. The insect was initially in saline at pH 7.2 and at time 0 minutes this was replaced by saline at pH 2 and after 13.5 minutes by saline at pH 12. In the acid pH, the intensity of the fluorescence gradually declined, only to increase again in the alkaline pH. (B) Graph of the changes in intensity measured from digital images, such as those shown in (A). Red squares and lines: summed fluorescence across pixels, normalised, within an outline approximately 1 mm long drawn to cover the brightest part of the pleural arch. Black filled circles: normalised mean maximum fluorescence intensity ± 1 standard deviation recorded along 10 lines approximately 0.55 mm long and 0.1 mm apart, orthogonal to the long axis of the pleural arch. Blue circles: background fluorescence assayed as the mean minimum intensity ± 1 standard deviation from the same lines.

The intense blue fluorescence had the general excitation and emission characteristics of the protein resilin. To provide further evidence that the fluorescence is from resilin, we analysed its properties at acidic and alkaline pH, which is known to affect the fluorescence of resilin [25,28,31]. The intensity of fluorescence was measured from digital images taken at a constant exposure from a 1-mm long region that encompassed most of the fluorescence as viewed from the side (Figure 5). The insect was initially placed in saline with a pH of 7.2 and once repeated measurements showed that the intensity of fluorescence was consistent, the saline was replaced by an acidic saline with a pH of 2. After 13 minutes in this saline, the intensity of the fluorescence had progressively declined (Figure 5A). This saline was then replaced with one of pH 12 and over the next 30 minutes, the intensity of the fluorescence gradually increased until it surpassed the intensity at the start of the experiment (Figure 5A). Neither the decline in the fluorescence nor its subsequent recovery had fully stabilised during the times allowed here, perhaps due to the time needed for penetration into this large structure, or the presence of other fluorescent material.

Three sets of measurements were made to quantify these changes (see Methods and Figure 5B). First, the summed fluorescence across pixels within the defined area was normalised and then plotted to give a measure of integrated density of the fluorescence. Second, the intensity of the normalised, mean, maximum fluorescence and ± 1 standard deviation (SD) was recorded on 10 lines approximately orthogonal to the long axis of the structure. Third, background fluorescence on the same scale was assayed as the mean minimum intensity ± 1 SD obtained from the same lines. The profiles confirmed that the fluorescence declined by almost one half in the acidic saline and then reversibly recovered in alkaline saline, finally to exceed, in places, the intensity first recorded in the neutral saline (Figure 5B).

### Partial emission spectrum of the blue fluorescence

**Figure 6:**
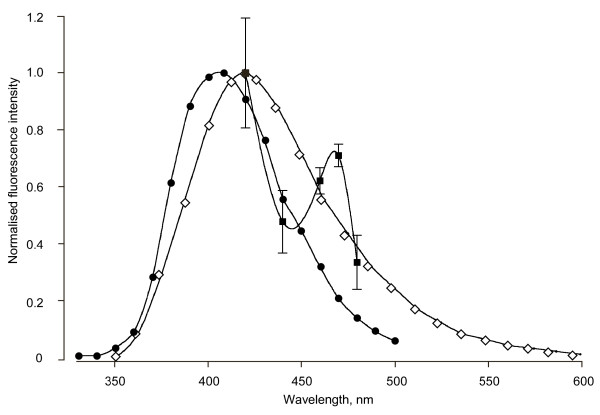
**Partial emission spectrum of the fluorescence**. The pleural arches were measured in six *Aphrophora *(filled squares ± 1 standard deviation) using five accessory interference filters with peaks falling within the 413–483 nm pass band of the Semrock emission filter. These measurements are compared with the known emission spectra of native resilin (diamonds, from Andersen [25]) and synthesised dityrosine (filled circles, from Malencik et al. [27]).

The emission spectrum of the blue fluorescence was averaged from six pleural arches at five wavelengths between 420 and 480 nm limited by the presence of the Semrock emission filter. The resulting partial spectrum (Figure 6) declined from the maximum near 420 nm, expected from the spectra of resilin and its chief determinants, dityrosine and trityrosine [25,27]. The spectra obtained, however, consistently revealed a peak near 460–470 nm not characteristic of resilin nor the tyrosine derivatives. This suggests again that a second blue-fluorescing component that is not resilin may be present in a pleural arch, possibly the same component implicated by the fluorescence that remained at pH 2 (Figure 5).

## Deformation of the pleural arches during muscle contraction

**Figure 7:**
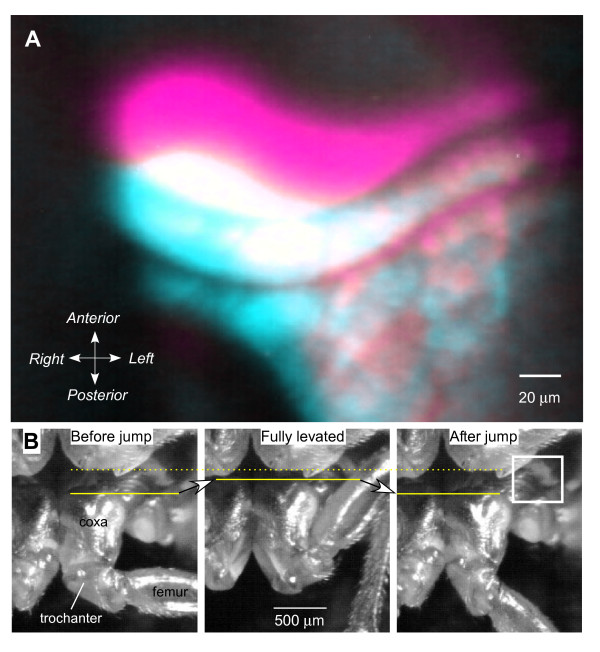
**Distortion of the pleural arches in jumping**. (A) Movement of the coxal end (see box in right image of B) of the pleural arch of the left hind leg when the left trochanteral depressor muscle was stimulated. Digital images under ultraviolet illumination at the start (cyan) and end (magenta) of the stimulation are superimposed. The colour changes were made in Photoshop, so that areas of overlap between the two images appear white. (B) Frames from a natural jump captured at 1000/second; the left frame was taken before the jump, the middle one after a 2-second long contraction of the trochanteral muscles, and the one on the right after the jump. The prolonged contraction results in a forward movement of the coxae (arrow and lines), which is reversed when the hind legs depress and extend in a jump.

To determine whether a pleural arch including its parts that contain resilin might move or be distorted during contractions of the trochanteral depressor muscles that power a natural jump, we carried out the following analysis. In an intact animal, the large thoracic parts of this muscle were stimulated by implanted electrodes. A sequence of stimuli (see Methods) reliably caused a sustained contraction of the muscle that resulted in a distortion and forward movement of the coxal end of the fluorescent pleural arch on the stimulated side (Figure 7A). This movement could be readily observed through the microscope, although the start of the stimulus sequence and the camera exposure could only be synchronised manually so that it was rarely possible to photograph the full extent of the movement. The mean distance moved that was recorded in this way was 19.1 ± 2.5 μm (*N *= 13, range 10–40 μm). In natural jumping where the two trochanteral depressor muscles moving both hind legs contract upon receipt of a tightly synchronised sequence of spikes to both, the coxal end of the bow moves forwards by 100 μm [6] (Figure 7B). The local electrical stimulation used here could at best activate only the thoracic parts of the muscle on one side.

As the posterior coxal end of the pleural arch moves forward while it seems that its anterior end stays fixed, this movement amounts to a compression of the bow with some bending. Ignoring any bending, a 100-μm movement in the 1084-μm long part of the pleural arch containing resilin, amounts to a linear compression of about 9%.

## Energy storage in the pleural arches

A pleural arch is a composite structure containing approximately 80% (by cross-sectional area) chitinous cuticle, some sclerotised but the majority translucent, 20% resilin and possibly other unknown proteins. How much of the total energy demands of jumping can be met by the two major components of the pleural arches?

First, we consider only the resilin part of a pleural arch. If this resilin is loaded in pure compression, then the elastic potential energy *U*_*e *_stored in one pleural arch is given by the following equation:

(1) $$Energy(U_{e})=\frac{EA}{2L}\delta L^{2}$$

where: *E *is Young's modulus for resilin; *A *is the cross-sectional area of the resilin part of the pleural arch; *L *is the resting length of the resilin part of the pleural arch; δ*L *is the change in length of the resilin under full compression during a jump.

The modulus (*E*) for resilin ranges from 3.0 × 10^5 ^to 3.0 × 10^6 ^Pa [37]. The cross-sectional area (*A*) of the resilin part of the pleural arch is 64 × 10^3 ^μm^2 ^and its resting length (*L*) is 1024 μm. The change in length (δ*L*) is 100 μm as measured from high speed images of the movements of the thoraco-coxal joint during a natural jump (data in [6]) (Figure 7). Assuming the length change is pure compression, the maximum energy storage capacity *U*_*e *_of the resilin is thus 0.9 μJ for one pleural arch, or 1.8 μJ for both. This is only 2% of the 88 μJ necessary to generate an average jump, and 1% of the 164 μJ necessary to generate the best jumps. Moreover, this calculation assumes that all of the strain is compressive, and thus overestimates the stiffness and energy capacity of the resilin part of the pleural arch. More realistically, an arch can compress, shear and bend, and would thus be less stiff than our estimate. In reaction to bending, the resilin part of the pleural arch would be about a tenth of the stiffness and would thus store a tenth of the energy as it would if it were in pure compression [38] (see the Additional file 1 for calculations of stiffness of a bending beam). Even with assumptions that overestimate the amount of energy stored in the resilin, the resilin alone is insufficient to store the energy required for a jump.

Second, we consider the chitinous cuticular part of the pleural arch. A whole pleural arch has a cross-sectional area (*A*) of 3.5 × 10^5 ^μm^2^, and a length (*L*) of 1500 μm. The change in length (δ*L*) is 100 μm as above. Assuming the length change is pure compression, then, via Equation (1), the chitinous cuticle of the two pleural arches would have to have a Young's modulus of 3.8 × 10^7 ^Pa to store 88 μJ for an average jump, or 7.0 × 10^7 ^Pa to store 164 μJ of energy for a maximum jump. The arch, however, can bend and shear as well. As the arch has a much greater depth (500 μm) than the resilin part (127 μm), it will not lose as much stiffness if allowed to bend and shear; it is approximately 30% as stiff if allowed to bend as it is in pure compression (see the Additional file 1 for calculations). The chitinous cuticle of the pleural arch would, therefore, need to have a modulus of 1.3 × 10^8 ^to 2.3 × 10^8 ^Pa to store the energy for jumps, well within the range for insect chitinous cuticle of 1 × 10^7 ^to 3 × 10^10 ^Pa [37]. These calculations indicate that the chitinous cuticle of the pleural arches stores most of the energy necessary to power a jump.

## Discussion

Froghoppers are now recognised as the champion insect jumpers, with one of the subjects studied here, *Philaenus*, generating a force at take-off that is over 400 times its body mass, with the other, *Aphrophora*, not far behind [5,32]. They achieve this prowess by apportioning 11% of their body mass to two trochanteral depressor muscles in the metathorax that power the catapult-like jump by the short hind legs [5,36]. Force is developed slowly by these muscles when driven by a motor pattern in which motor spikes to both hind legs are tightly synchronised [36]. We have shown here that the energy generated by these prolonged contractions is stored in distortions of an enlarged pair of pleural arches that are part of the internal skeleton. These are composite structures of chitinous cuticle and resilin, which was identified by its brilliant pH-dependent fluorescence and its limited spectral signature [18,28]. Larval froghoppers that do not jump lack resilin in their pleural arches. When the main trochanteral depressor muscle of an adult was stimulated with a sequence of pulses matching the pattern of their motor spikes recorded during a natural jump, the posterior end of the fluorescent pleural arch moved forward, was compressed by about 9% and was also bent. Calculations (see Additional file 1) indicate that all the energy required for jumping could be stored in the pleural arches with the resilin and cuticular parts serving different functions. The chitinous cuticle of the pleural arches may itself be of mixed types.

## The fluorescence in the pleural arches is from resilin

Three lines of evidence indicate that the fluorescence we describe for the pleural arches of froghoppers is from resilin.

First, the tarsal pads of the cockroach *Periplaneta*, which Neff et al. [31] argue contain resilin, also fluoresced bright blue with our optical system.

Second, quantification of the pH dependence of the fluorescence strongly suggests that it is generated by resilin. Most parts of a pleural arch completely lost their blue fluorescence at pH 2, although overall the integrated measurements of fluorescence fell only to about half. It is difficult to compare this result quantitatively with the results of Neff et al. [31] because they present numerical results only as a difference curve after subtracting the fluorescence observed at low pH, so the baseline is unknown. In the excitation band above 350 nm available through the Semrock filter set we used, absorption by the dityrosine or trityrosine residues will be effectively zero at acid pH [25,27]. The considerable fluorescence remaining even at pH 2 therefore, cannot come from the low pH form of resilin [31], but must emanate either from resilin that is somehow protected from the bathing solution, or from some other fluorescent compound. The fluorescent part of a pleural arch is large so that diffusion times could be a factor, but the exponential-like reduction in fluorescence had almost reached its asymptote at the times used (see Figure 5B). The onset of the effect was nonetheless very slow compared with that experienced by Neff et al. [31], who report that pH effects on resilin in the relatively small tarsal pads of cockroaches require 'no special incubation times' and were complete within 5 minutes, again arguing that diffusion times were a factor here.

Third, the declining spectral signature of the fluorescence from the pleural arch obtained from 420 to 480 nm fits the expectation for resilin, but contains what appears to be evidence for an additional fluorescent compound emitting at 460 to 470 nm. While this provisional result is encouraging, the determination needs to be extended over a wider range of wavelengths to allow a more stringent comparison with the known spectral emission of resilin [25]. This result does, however, suggest additional complexity and further emphasises the composite nature of the pleural arches of froghoppers.

The fluorescence emission of resilin at all pH values is highest near 420 nm, while the excitation maxima of dityrosine and trityrosine and presumably resilin lie at 317 to 323 nm at neutral and high pH, but absorption continues towards the near UV [25]. Comparing our results with previous results [31] requires consideration of the possible effects of the different filter sets and cameras that were used. The Semrock set we used should be superior in isolating the blue fluorescence of resilin from longer-wavelength fluorescent compounds by virtue of the limited blue band pass of the Semrock exit filter, in contrast to the long-pass Nikon UV-2A filter set used by Neff et al. [31]. It will also completely eliminate contaminating UV absorption by the low pH 2 form of resilin encountered in pH tests, unlike the UV-2A set. The Nikon set should be about twice as effective at exciting resilin at neutral pH, but this was not relevant in our study because, depending on the objective lens used, the excitation beam had to be attenuated by 25% or 6% with an accessory neutral filter, to avoid saturating the blue channel of the camera where all the emission collected. Both at the input and output, therefore, the filter set used here should be more discriminating in isolating the fluorescence of resilin than the UV-2A set.

## Structures storing energy

Pleural arches are an integral feature of the internal thoracic skeleton of many insects. In fulgorids (another group in the Hemiptera, Auchenorrhyncha), which also jump, the arch is also enlarged [33,34]. Fleas (Siphonaptera) rely on the pleural arches to make the thorax rigid when powering their jumps by contractions of trochanteral depressor muscles in the thorax [22]. In fleas, it is suggested that the energy for jumping is stored in small pads of resilin at the dorsal end of the pleural arches [21]. Fleas which parasitise the largest and fastest mammalian hosts have larger amounts of resilin [22]. A loss or reduction of the pleural arches is correlated with a reduction in jumping abilities, but some species of flea lacking pleural arches can still jump.

In proportion to the size of the body, the resilin in the metathoracic pleural arches of froghoppers is huge. The 1-mm long fluorescent region in a single pleural arch of an adult 10 mm long and 28 mg in mass, gives a volume-per-body-mass ratio of 2.3 mm^3^/g. In the flea that has a mass of 4.5 × 10^-4 ^g (0.45 mg), one resilin pad has a volume of 1.04–1.75 × 10^-4 ^mm^3 ^[21], so that the volume/mass ratio is 0.23 mm^3^/g, or 10 times smaller than that of a froghopper. Expressed in the same way, the resilin in a froghopper is orders of magnitude larger than the resilin in a cockroach leg [31], or in a cicada tymbal, neither of which are used in the rapid release of large amounts of elastic energy [39].

## Conclusion

There is a rather pervasive assumption, prompted in part by the interpretation of the way fleas jump [21-23], that resilin alone is sufficient to meet the large power demands of actions such as jumping. Chitinous cuticle was thought to be used for energy storage only when the deformation was limited, while resilin was used when the larger, reversible deformations were needed [28]. We were, therefore, surprised initially that the resilin part of the pleural arches of froghoppers can do so little towards supplying the power surge to the legs required for jumping. To estimate the capacity of the resilin in fleas to store energy, Bennet-Clark and Lucey [21] assumed, nevertheless, that the resilin would be subjected to a strain of over 100%, although the excursion was not measured. If, however, the strain was only in the same range (approximately 9%) as that measured in froghoppers, most of the energy storage for jumping in fleas remains unexplained. Moreover, the stiffness of resilin, as gauged by its relatively low Young's modulus [37], is much too small to store the energy required for jumping in froghoppers. By contrast, several different types of chitinous cuticle have enough stiffness to store the requisite energy for the displacement observed here (see the Additional file 1).

It therefore seems likely that resilin is used in two distinctly different ways by insects. The first involves its use as an energy buffer in rhythmically active, fast mechanical movements, such as those of the wings during flight or the tymbals in cicadas for generating sound [19]. The almost perfect elastic recovery of resilin and its extreme resistance to mechanical fatigue means that it can return nearly all of the power put into it for the next cycle of movement [28]. The second role, relevant here, is in providing a flexible material that is combined with the stiffer chitinous cuticle in a composite structure. The resilin could also ensure that the original shape of the body is rapidly and fully restored after a jump.

The combination of resilin and chitinous cuticle in the pleural arches may work like a composite bow used in archery. Composite bows made from materials with different properties have three advantages over simple bows made of just one material [40] that are directly pertinent to their use by froghoppers. First, composite bows lose significantly less energy to vibration than do simple bows. This would allow froghoppers to transfer energy more effectively from the elastic energy store to its hind legs. Second, the mechanical properties of composite bows change significantly less with repeated use. This would allow froghoppers to generate repeatedly jumps that are precise and powerful even after repeated loading of the pleural arches in preceding jumps. Third, composite bows can be kept strung for long periods of time without losing their mechanical properties. This would allow froghoppers to keep their pleural arches 'tensed' and ready for a jump, without the tension creeping, or the mechanical properties changing. Resilin would be particularly effective at preventing creep because of its ability to withstand large strains over a long time without any measurable creep [28]. Behavioural observations show that froghoppers frequently hold their hind legs fully levated and cocked (and thus with the pleural arches tensed) ready for jumping and that they do not use them in walking movements on horizontal surfaces [32].

It is not yet known if the pleural arches in froghoppers are laminated or form a more integrated composite that might have greater strength per unit mass and greater resistance to vibration. This would allow large areas of shear strain in the matrix and greater shock absorption during energy release. Furthermore, by distributing stresses around areas of weakness, a resilin-chitinous cuticle composite would mitigate against structural failure and lessen the likelihood of cracking, leading to improved fatigue life.

Our results suggest that structures storing energy for activities such as jumping should be composites combining the stiffness of chitinous cuticle and the resilience of resilin. Two predictions then flow about the structures for storing energy in other insects. For example, in fleas where compression of resilin alone is proposed as the energy store [21], a contribution from a stiffer structure that needs less compression may be necessary to store sufficient energy for a jump. Conversely, in locusts, the cuticular semi-lunar processes, which store half the force for jumping [13], may be unable to withstand the strains experienced without the participation of resilin, which has not been described in these structures.

## Methods

### Animals

Adult froghoppers (spittle bugs) *Aphrophora alni *(Fallén, 1805) with a body length in females of 9.7 mm ± 0.24 SD (*N *= 18) and males of 9.1 mm ± 0.38 (*N *= 15) were collected in August and September 2007 from low vegetation bordering woodland near Halifax, Nova Scotia, Canada. This species was distinguished from the similar endemic species *A. quadrinotata*, based mainly on body length and the ratio of head length to pronotum length ([41] and personal communication). Unless otherwise stated, all the data presented are from this species. Some adults of a smaller froghopper, *Philaenus spumarius *(Linnaeus, 1758), which also jumps prodigiously, and its various larval stages, which are confined to a frothy mass on a host plant and do not jump, were collected from the same sites. Both species were either examined on the day they were caught or were maintained at 4–5°C for a few days. They both belong to the order Hemiptera, sub-order Auchenorrhyncha, superfamily Cercopoidea, family Aphrophoridae.

### UV microscopy

Live intact specimens were restrained ventral surface up, or on their side in a Petri dish with a floor of Sylgard, and placed on the stage of an Olympus BX51WI compound microscope. A Leica MZ18 stereo microscope with an attached drawing tube was also used to reveal the structure of a pleural arch. When the metathorax was dissected by removal of the ventral cuticle and muscle, the insect was covered with a saline developed for *Drosophila *[42]. The metathorax was viewed through an Olympus MPlan ×5/0.1 NA, or LUCPlanFLN ×20/0.45 NA objective lenses, under UV or white epi-illumination. Images were captured with a Leica EC3 digital camera and associated Leica software as colour (RGB) TIFF files. During each experiment, the camera gain and exposure time were kept fixed. UV light was provided by an Olympus U-LH100HGAPO, 100 W mercury arc, conditioned by a Semrock DAPI-5060B Brightline series (Semrock, Rochester, NY, US), high-brightness UV filter set with a sharp-edged (1% transmission limits) band from 350 to 407 nm. The resulting blue fluorescence emission was collected in a similarly sharp-edged band at wavelengths from 413 to 483 nm through a dichromatic beam splitter. The overall intensity of the exciter beam was attenuated with neutral density filters to match the particular objectives used. To photograph in the visible range by epi-illumination, two high-intensity, white light-emitting diodes (Phillips LumiLED type LXHL-NWE8) were placed above the preparation, projecting down at 30° to 40° incident angles from opposite sides of the objective. Images captured at the same focal planes under UV and visible light were superimposed using Photoshop (Adobe).

### Properties of resilin

To analyse the properties of the blue fluorescence, the pH of the bathing saline was changed from its normal value of 7.2 to pH 2 with 2 M hydrochloric acid, and to pH 12 with 2 M sodium hydroxide. Changes in fluorescence were captured as images and quantified using the programme ImageJ  by measuring along each of 10 predefined sampling lines about 550 μm long and 100 μm apart and orthogonal to the long axis of the main fluorescent structure. Each line first sampled the non-fluorescent background of the pleural arch, then the bright fluorescence, and finally back into the surrounding non-fluorescent region. The maximum-minimum measurements, therefore, gave the dark background and the fluorescence of the brightest part of the pleural arch on each line. These values were then averaged over the 10 lines to give a measure of the relative fluorescence and the general background fluorescence. The summed intensity signal across all pixels within the outline of the fluorescing structure was calculated with the 'integrated density' function in ImageJ. Calibrated neutral density filters were used to show that the blue channel of the Leica camera had a satisfactorily linear input-output relationship above threshold, provided that channel saturation was avoided by inserting accessory neutral filters into the excitation path.

To characterise the blue fluorescence further, its spectral signature was measured between 420 and 480 nm using five narrow-band interference filters (bandwidth at half-maximum 8–10 nm), all having maxima lying within the 413–483 nm pass band of the Semrock blocking filter, which was left in place. The pleural arch was photographed with each interference filter in turn interposed in the light path to the camera, sometimes with additional neutral density filters. The mean intensity of fluorescence over the brightest parts of the digital images was averaged five times with ImageJ. The mean values were then adjusted to compensate for the unequal transmittances of the interference and neutral filters as determined with a Varian DMS90 spectrophotometer, and for the relative spectral response of the blue channel of the camera (information provided by Leica Microsystems, Cambridge, UK). The intensity of the blue fluorescence varied by almost a factor of two between specimens and was, therefore, normalised before the results obtained at each wavelength were averaged for comparison with the emission spectrum of resilin [25].

### Jumping simulation

To determine if the fluorescent structures in the thorax might be moved and distorted during the sequence of muscle contractions that precede the rapid movement of the hind legs in a jump, two 75-μm diameter silver wires, insulated except at their tips, were inserted through the ventral thoracic cuticle and into the trochanteral depressor muscle of the left hind leg of an intact insect. The pattern of motor spikes in this muscle used by a froghopper during natural jumping movements has been determined [36]. This pattern was simulated by applying a 1-second long sequence of stimuli at a frequency of 40 Hz, a duration of 0.5 ms, and an amplitude of 0.4 to 2 V, generated by a Grass SD-9 pulse generator. The resulting effects of the evoked muscle contraction were recorded under UV illumination before and then during stimulation. The range of movement captured photographically was usually smaller than that observed while watching through the microscope or the screen of a computer storing the images, because the camera exposure could only be linked manually to the stimulus sequence.

The data on UV fluorescence are based on 10 adult *Aphrophora*, with comparable fluorescent structures seen in seven *Philaenus*, and nine larval *Philaenus*. The data on the internal structure of the pleural arch are based on a further 20 adult *Aphrophora*.

## Abbreviations

SD: standard deviation; UV: ultraviolet.

## Authors' contributions

MB performed the dissection and anatomy work. MB and SRS carried out the UV microscopy studies and muscle stimulation experiments. SRS analysed the partial emission spectrum of the resilin. GPS calculated the potential energy storage of the resilin and provided the supplementary calculations. All the authors worked on and approved the final manuscript.

## Supplementary Material

Additional file 1 **Appendix. Estimate of the stiffness of the resilin part of the pleural arch.** Click here for file

## References

[B1] Zajac FE (1989). Muscle and tendon: properties, models, scaling, and application to biomechanics and motor control. Crit Rev Biomed Eng.

[B2] Vogel S (2005). Living in a physical world III. Getting up to speed. J Biosci.

[B3] Alexander RM, Bennet-Clark HC (1977). Storage of elastic strain energy in muscle and other tissues. Nature.

[B4] Gronenberg (1996). Fast actions in small animals: springs and click mechanisms. J Comp Physiol [A].

[B5] Burrows M (2003). Froghopper insects leap to new heights. Nature.

[B6] Burrows M (2006). Morphology and action of the hind leg joints controlling jumping in froghopper insects. J Exp Biol.

[B7] Burrows M (1969). The mechanics and neural control of the prey capture strike of the Mantid shrimps *Squilla *and *Hemisquilla*. Z Vgl Physiol.

[B8] Patek SN, Nowroozi BN, Baio JE, Caldwell RL, Summers AP (2007). Linkage mechanics and power amplification of the mantis shrimp's strike. J Exp Biol.

[B9] Gronenberg W (1995). The fast mandible strike in the trap-jaw ant *Odontomachus*. I. Temporal properties and morphological characteristics. J Comp Physiol [A].

[B10] Godden DH (1975). The neural basis for locust jumping. Comp Biochem Physiol.

[B11] Heitler WJ, Burrows M (1977). The locust jump. I. The motor programme. J Exp Biol.

[B12] Burrows M (1995). Motor patterns during kicking movements in the locust. J Comp Physiol [A].

[B13] Bennet-Clark HC (1975). The energetics of the jump of the locust *Schistocerca gregaria*. J Exp Biol.

[B14] Burrows M, Morris G (2001). The kinematics and neural control of high speed kicking movements in the locust. J Exp Biol.

[B15] Evans MEG (1972). The jump of the click beetle (Coleoptera: Elateridae) – a preliminary study. J Zool Lond.

[B16] Evans MEG (1973). The jump of the click beetle (Coleoptera, Elateridae) – energetics and mechanics. J Zool Lond.

[B17] Kaschek N (1984). Vergleichende Untersuchungen über Verlauf und Energetik des Sprunges der Schnellkäfer (Elateridae, Coleoptera). Zool Jb Physiol.

[B18] Weis-Fogh T (1960). A rubber-like protein in insect cuticle. J Exp Biol.

[B19] Bennet-Clark HC (1997). Tymbal mechanics and the control of song frequency in the cicada *Cyclochila australasiae*. J Exp Biol.

[B20] Fonseca PJ, Bennet-Clark HC (1998). Asymmetry of tymbal action and structure in a cicada: a possible role in the production of complex songs. J Exp Biol.

[B21] Bennet-Clark HC, Lucey ECA (1967). The jump of the flea: a study of the energetics and a model of the mechanism. J Exp Biol.

[B22] Rothschild M, Schlein J (1975). The jumping mechanism of *Xenopsylla cheopis*. Exoskeletal structures and musculature. Philos Trans R Soc Lond B Biol Sci.

[B23] Rothschild M, Schlein J, Parker K, Neville C, Sternberg S (1975). The jumping mechanism of *Xenopsylla cheopis*. III. Execution of the jump and activity. Philos Trans R Soc Lond B Biol Sci.

[B24] Sannasi A (1969). Resilin in the cuticle of click beetles. J Georgia Entomol Soc.

[B25] Andersen SO (1963). Characterization of a new type of cross-linkage in resilin, a rubber-like protein. Biochim Biophys Acta.

[B26] Andersen SO (1964). The cross links in resilin identified as dityrosine and trityrosine. Biochim Biophys Acta.

[B27] Malencik DA, Sprouse JF, Swanson CA, Anderson SR (1996). Dityrosine: preparation, isolation, and analysis. Anal Biochem.

[B28] Andersen SO, Weis-Fogh T (1964). Resilin. A rubberlike protein in arthropod cuticle. Adv Insect Physiol.

[B29] Jensen M, Weis-Fogh T (1962). Biology and physics of locust flight. V. Strength and elasticity of locust cuticle. Philos Trans R Soc Lond B Biol Sci.

[B30] Mindrinos MN, Petri WH, Galanopoulos VK, Lombard MF, Margaritis LH (1980). Crosslinking of the *Drosophila *chorion involves a peroxidase. Rouxs Arch Dev Biol.

[B31] Neff D, Frazier SF, Quimby L, Wang R-T, Zill S (2000). Identification of resilin in the leg of cockroach, *Periplaneta americana: *confirmation by a simple method using pH dependence of UV fluorescence. Arthropod Struct Dev.

[B32] Burrows M (2006). Jumping performance of froghopper insects. J Exp Biol.

[B33] Sander K (1957). Bau und Funktion des Sprungapparates von *Pyrilla perpusilla *Walker (*Homoptera – Fulgoridae*). Zoology (Jena).

[B34] Heilig S, Sander K (1986). Zahnradsektoren zur Koordination der Sprungbeine – eine lavale Synapomorphie der fulgoromorphen Zikaden (Homoptera, Cicadina, Fulgoroidea). Zool Jb Syst.

[B35] Gorb SN (2004). The jumping mechanism of the cicada *Cercopis vulnerata *(Auchenorrhyncha, Cercopidae): skeleton-muscle organisation, frictional surfaces, and inverse-kinematic model of leg movements. Arthropod Struct Dev.

[B36] Burrows M (2007). Neural control and co-ordination of jumping in froghopper insects. J Neurophysiol.

[B37] Vincent JFV, Wegst UGK (2004). Design and mechanical properties of insect cuticle. Arthropod Struct Dev.

[B38] Boresi AP, Schmidt RJ, Sidebottom OM (1993). Advanced Mechanics of Materials.

[B39] Young D, Bennet-Clark H (1995). The role of the tymbal in cicada sound production. J Exp Biol.

[B40] Miller R, McEwen E, Bergman C (1986). Experimental approaches to ancient near eastern archery. World Archaeol.

[B41] Hamilton KGA (1982). The Insects and Arachnids of Canada Part 10 The Spittlebugs of Canada Homoptera: Cercopidae.

[B42] Stewart BA, Atwood HL, Renger JJ, Wang J, Wu CF (1994). Improved stability of *Drosophila *larval neuromuscular preparations in haemolymph-like physiological solutions. J Comp Physiol [A].

