# Supplementary material for: Resilin and chitinous cuticle form a composite structure for energy storage in jumping by froghopper insects
Source: BMC Biol. 2008 Sep 30;6:41. doi: 10.1186/1741-7007-6-41 (PMC2584104; doi:10.1186/1741-7007-6-41)
Supplement: Additional file 1 — Appendix. Estimate of the stiffness of the resilin part of the pleural arch. [file 1741-7007-6-41-S1.doc]

## Estimate of the stiffness of the resilin part of the pleural arch

The resilin part of a pleural arch was approximated as a curved beam with the following initial dimensions (undeformed):

The stiffness of the curved beam is calculated by summing the compliance of the beam in response to bending (*C_b_*), shear (*C_s_*), and compression (*C_c_*), and then taking the reciprocal of the sum of the compliances.

The net compliance (*C_t_*) of the entire structure can be calculated as:

(A1)

Each compliance was calculated by summing the bending strain, shear strain and compressive strain caused by applying a unit force to the ends of the beam.

The bending compliance of the structure (*C_b_*) is calculated by summing the strain caused by the bending moment on the beam (Chapter 9 in [38]):

(A2)

where *L* is the length of the beam (1084 μm), *E* is Young’s modulus of resilin (3×10^6^ Pa; Vincent and Wegst [37]), *A* is the cross-sectional area of the beam (64,008 μm^2^), *A*_m_ is the location of the neutral axis (the axis of zero strain) of a curved beam, and ρ is the beam’s initial radius of curvature (between 700 and 1200 μm, see Figures 1 and 3). For a beam with this square cross-section *A*_m_ is equal to:

(Chapter 9 in [38]) (A3)

The shear compliance (*C_s_*) is calculated by integrating the shear strain caused by a unit force (Chapter 9 in [38]):

(A4)

where *G* is the bulk modulus, which for iso-volumetric biological tissue is approximately *E*/3.

The compression compliance (*C_c_*) is calculated by integrating the normal strain caused by a unit force:

(A5)

The stiffness of the entire structure can then be calculated by taking the reciprocal of the sum of the compliances:

(A6)

The above integrals can then be evaluated for curved beams with different radii of curvature (ρ).  This allows us to calculate the stiffness for the resilin part of a pleural arch having differing initial curvatures.

The smaller the initial radius of curvature, the less stiff the beam becomes. As the radius of curvature approaches infinity, the stiffness approaches , yielding 188 N/m, which is the stiffness of the resilin part of a pleural arch in pure compression.

As the resilin part of the pleural arch becomes more curved, the less stiff it becomes, and consequently the less energy it can store. Its stiffness in pure compression is 188 N/m, while its stiffness with a radius of curvature of 0.7 to 1.2 mm is only about 10% of this (13-33 N/m). A pleural arch with this range of curvature, therefore, stores only about 10% of the energy of a straight one (from Equation (1) in the text).

## Estimate of the stiffness of the cuticular part of the pleural arch

To estimate the loss of stiffness of the pleural arch from bending, the above calculations were repeated for the cuticular part of the pleural arch with the following dimensions:

From Equations (A1)-(A5), the ratio of the net stiffness () to the stiffness of an unbent section loaded in pure compression () can be calculated as a function of radius of curvature (ρ).

With a radius of curvature of 0.7 to 1.2 mm, the cuticular section of a pleural arch is only approximately 30% as stiff as it would be if it were a straight beam loaded in pure compression.
